# A Review of Canadian Cancer-Related Clinical Practice Guidelines and Resources during the COVID-19 Pandemic

**DOI:** 10.3390/curroncol28020100

**Published:** 2021-02-25

**Authors:** Eliya Farah, Rami Ali, Parker Tope, Mariam El-Zein, Eduardo L. Franco

**Affiliations:** Division of Cancer Epidemiology, Gerald Bronfman Department of Oncology, McGill, University, 5100 Maisonneuve Blvd West, Suite 720, Montreal, QC H4A 3T2, Canada; eliya.farah@mail.mcgill.ca (E.F.); rami.ali2@mail.mcgill.ca (R.A.); parker.tope@mcgill.ca (P.T.); eduardo.franco@mcgill.ca (E.L.F.)

**Keywords:** COVID-19, cancer care, cancer control, clinical practice guidelines, cancer resources

## Abstract

(1) Background: Preventive measures taken in response to the coronavirus disease 2019 (COVID-19) pandemic have adversely affected an entire range of cancer-related medical activities. The reallocation of medical resources, staff, and ambulatory services, as well as critical shortages in pharmaceutical and medical supplies have compelled healthcare professionals to prioritize patients with cancer to treatment and screening services based on a set of classification criteria in cancer-related guidelines. Cancer patients themselves have been affected on multiple levels, and addressing their concerns poses another challenge to the oncology community. (2) Methods: We conducted a Canada-wide search of cancer-related clinical practice guidelines on the management and prioritization of individuals into treatment and screening services. We also outlined the resources provided by Canadian cancer charities and patient advocacy groups to provide cancer patients, or potential cancer patients, with useful information and valuable support resources. (3) Results: The identified provincial guidelines emphasized *cancer care* (i.e., treatment) more than *cancer control* (i.e., screening). For cancer-related resources, a clear significance was placed on *knowledge & awareness* and *supportive resources*, mainly relating to mental health. (4) Conclusion: We provided a guidance document outlining cancer-related guidelines and resources that are available to healthcare providers and patients across Canada during the COVID-19 pandemic.

## 1. Introduction

The coronavirus disease 2019 (COVID-19) pandemic has been a major threat globally to public health. Balancing the risk of exposure to SARS-CoV-2 infection with that of oncological complications has necessitated a logistical transformation in healthcare provision across hospitals, clinics, and centres administering cancer screening and treatment services. In response to the unprecedented nature of the COVID-19 pandemic and its consequential, abrupt disruptions to many routine and elective medical services, public health agencies have issued cancer-related clinical practice guidelines (CPGs) and/or adapted directives based on expert opinions and consensus decisions to effectively manage and sustain cancer care and control services. These CPGs have provided guidance for triaging patients with cancer into treatment, and for prioritizing those under suspicion of cancer for screening and diagnostic services. The pandemic has also brought unparalleled psychological, social, and financial challenges to patients with cancer. Such challenges were compounded by patients’ vulnerability to infection and its clinical consequences, which increase the likelihood of admission to intensive care units, and fatality [1]. 

In this review, we provide a guidance document based on our assessment of cancer-related CPGs and resources in Canada for the COVID-19 era. It guides physicians to CPGs issued by provincial and territorial public health agencies in Canada, and guides patients with cancer, and those under suspicion of cancer, to various support resources offered by Canadian cancer charities and patient advocacy groups, hereafter referred to as organizations. 

## 2. Materials and Methods

### 2.1. Search Technique and Data Collection

We identified, for all ten provinces and three territories in Canada, the respective governmental body responsible for overseeing cancer-related CPGs. We then screened each public health agency’s website for pre- and during COVID-19 cancer-related CPGs. These are presented in Table 1. If guidelines were unavailable on the website, we contacted the agency, either by email or telephone. Those that did not release CPGs were asked whether they were adopting guidelines from other provincial or territorial agency(ies).

Regarding cancer-related resources, we consulted the Canadian Cancer Society and Canadian Revenue Agency for a list of non-profit organizations, foundations, support groups, and community care groups. To be included in this review, these organizations needed to (1) be Canadian-based; international societies with Canadian branches were excluded, and (2) provide resources and recommendations directly related to the COVID-19 pandemic and/or redirect users to external sources for such information.

### 2.2. Data Synthesis

**Figure 1:**
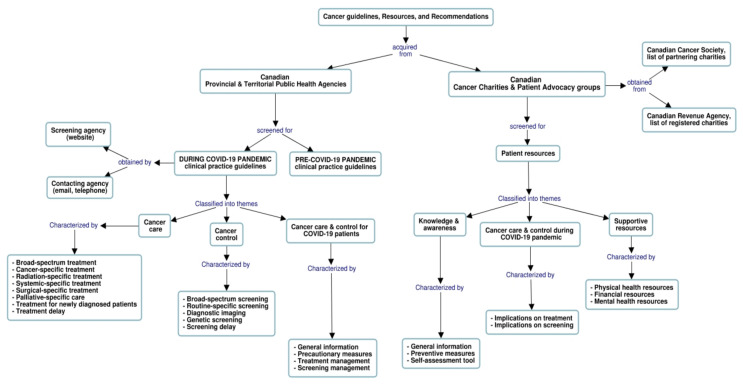
Concept mapping of search techniques, data collection process, and descriptive components.

We used “Cmaptools” [25,26] to generate a graphical concept map of the methods used to identify and describe the retrieved provincial and territorial guidelines provided in Table 1, resources, and recommendations for cancer care and control during the COVID-19 era. The concept map in Figure 1 illustrates the search techniques as well as data collection, characterization, and classification into themes and descriptive components. 

We classified the focus of the retrieved CPGs into three themes: *cancer care*, *cancer control*, and *cancer care & control for COVID-19 patients*. Each theme was further classified into various descriptive components to highlight major modifications to pre-existing guidelines and capture mitigation strategies employed to assist physicians in coping with the pandemic impacts. We derived these descriptive components by appraising the contents of the CPGs for key elements in relation to treatment and screening services as well as prioritization schemes. Table 2 provides, by theme, an overview of these components. 

Similarly, we grouped available cancer-related resources and recommendations into three themes: *knowledge & awareness*, *cancer care & control for cancer patients during COVID-19*, and *supportive resources*. We list in Table 3, by theme, their related descriptive components and characteristics. These components were established based on the results of a survey that was conducted by the Quebec Cancer Coalition to assess a series of unmet needs for patients with cancer during the COVID-19 pandemic [27]. In this survey, most respondents indicated that the continuity of their care has been affected by the pandemic, their mental health has worsened, and their medical appointments (e.g., imaging, follow-up, surgery) have been postponed or cancelled, in addition to other significant alterations and challenges in their cancer care. The reported quantitatively assessed challenges and qualitatively presented experiences of cancer patients informed the topics to be addressed and the establishment of the above-mentioned overarching resources.

The flowchart illustrates the methods used to identify and describe Canadian guidelines, resources, and recommendations for cancer care and control during the COVID-19 era. Information collected from multiple sources was classified into major themes and characterized accordingly (refer to Table 2 and Table 3 for a detailed description).

## 3. Results

### 3.1. Cancer-Related Clinical Practice Guidelines (CPGs)

Following the declaration of the COVID-19 outbreak as a global pandemic by the World Health Organization in March, the government of Canada rapidly responded by declaring a state of emergency across all provinces and territories [28]. By the end of April, the provincial public health agencies of British Colombia, Ontario, and Quebec issued a set of cancer-related CPGs, as shown in Table 1, to provide health professionals with guidance on modifications to oncology services. 

Table 4 lists the components of these CPGs by theme. Those issued by British Columbia Cancer Agency (BCCA), Cancer Care Ontario (CCO), and Quebec’s Ministère de la Santé et des Services Sociaux (MSSS) covered components across all themes. All CPGs included prioritization criteria to triage patients with cancer into treatment based on several factors. MSSS and CCO propose triage of patients into treatment priority levels (A, B, and C) based on the need and efficacy of treatment [20,22]. Alberta Health Services (AHS) [3] referred to guidelines developed by Kutikov et al., which considers the risk of progression with cancer care delay as a factor in triaging patients into priority levels (low, intermediate, and high risk) [29].

Factors such as urgency for treatment and ability of treatment to decrease risk of death, reduce morbidity, and maximize quality of life were used by BCCA to classify patients into four priority phases [7]. Phase 1 is assigned to individuals with emergencies and life-threatening conditions (e.g., spinal cord compression), whereas phase 4 is assigned to individuals with nonurgent treatments (e.g., palliative, and adjuvant therapy). Similarly, the Nova Scotia Health Agency (NSHA) proposes triaging patients into four “band” levels based on perceived urgency for the patient to receive treatment [17]. Band 1 refers to patients with life/limb/organ threatening conditions (e.g., malignancy with ongoing airway/swallowing compromise) which requires immediate treatment, while band 4 refers to individuals with conditions for which treatment delay will unlikely result in adverse oncologic outcomes (e.g., low risk prostate cancer). Age, comorbidities, tumour stage, type of treatment, as well as mental and physical ability were also considered as parameters in the decision-making process, but not sufficient for patient prioritization. Other factors include curative treatments with a high probability of success, center capacity, and staff availability in cancer centers.

Although all identified CPGs described prioritization criteria for deciding which patients are in most need of treatment, only MSSS, CCO, and BCCA, stratified these according to cancer site (e.g., skin) and tumor type (e.g., melanoma) [8,19,22]. Most provinces provided radiation-, systemic-, and palliative- specific guidelines, benchmarks for wait times for each of these target-treatments, and recommendations to manage treatments for newly diagnosed cancer patients. Furthermore, prioritization of surgical procedures was provided in CPGs issued by all agencies except NSHA and BCCA.

In regard to the *cancer control* theme, the MSSS, CCO, and BCCA—the only agencies that included cancer control in their CPGs—used different approaches for triaging screening services [7,20,22]. MSSS developed a prioritization system that assigns patients into three levels of clinical activity; minimal, moderate, and optimal, each pertaining to specific screening recommendations based on patients’ pre-determined risk of developing cancer [22]. CCO’s screening priority level A represents the highest level calling for immediate examination to determine a patient’s urgent treatment plan, level B assigns follow-up for additional screening or initiation of treatment plans based on abnormal screening results, and level C exemplifies all screening services that can be deferred for the entire pandemic [20]. Likewise, BCCA considered the risk of postponing a screening service when prioritizing individuals to stages A, B, C, and D, with stage A denoting little to no risk of tumour progression to patients if a screening test were to be postponed for eight weeks or until the pandemic is over [7]. Alternatively, stage D authorizes a screening procedure to be performed as a priority within a specified period (e.g., 24 h) as delays might pose serious health risks to an individual [7].

As for the third theme *cancer care & control for patients with COVID-19*, most provinces addressed mitigation measures and treatment management that were consistent across all CPGs; COVID-19 patients should not attend a clinic or receive cancer-related treatments. Patients who have already been infected with the virus are considered safe to undergo treatment only if they were asymptomatic and 14 days have elapsed since onset of symptoms [7,20,22]. None of the CPGs provided any guidelines/recommendations for screening management.

Overall, the most common cancer care and control modifications that were introduced during the pandemic to mitigate the risk of infection include: incremental integration of hypofractionation (i.e., dividing the total dose of radiation into large doses so that patients can complete their therapy faster) and/or accelerated fractionation for patients with cancer who require radiation therapy treatments; prioritizing oral anticancer drugs over intravenous chemotherapy and consideration of home-based infusion chemotherapy, when safely applicable; integration of telemedicine services (telephone or video consultations) to reduce clinic visits; providing alternative procedures to in-person screening, when available (i.e., stool DNA testing: Cologaurd for colon cancer); and encouraging home delivery of medications and online payments to minimize the risk of exposure to SARS-CoV-2.

As of this writing, no publicly available pre- or during- COVID-19 CPGs were found for all three territories of Canada (Yukon, Northwest Territories, Nunavut) and two provinces (Prince Edward Island and New Brunswick). According to public health agencies of Yukon and the Northwest Territories, no alterations to oncological practices were imposed by the pandemic due the lack COVID-19 cases in those territories. Through direct communication with the appropriate agency, we learned that Nunavut and provinces such as New Brunswick and Prince Edward Island have been referring to CPGs from British Columbia, Ontario, and Quebec. Even though the public health agencies of Saskatchewan and Newfoundland provided pre-COVID-19 guidelines, we were not able to identify whether these two provinces have issued cancer-related CPGs during the pandemic, despite multiple attempts to contact the agencies via email and telephone.

### 3.2. Cancer-Related Resources 

Contextualizing the COVID-19 pandemic within the cancer patient’s experience, some cancer organizations addressed how the pandemic has affected cancer treatment and screening in terms of treatment delays or appointment changes. On their COVID-19 webpages, organizations that recognize treatment alterations or delays encouraged patients with cancer to communicate with their physicians and care teams regarding treatment continuation. Organizations, such as the Quebec Cancer Coalition [27] or Colorectal Cancer Canada [30], that represent communities that have been diagnosed with cancers that are regularly screened for referred users to provincial guidelines concerning cancer screening. 

As shown in Table 5, of the 187 cancer organizations screened, 22 societies provided COVID-19 information specific to patients with cancer, each with varying levels of user engagement. Cancer organizations emphasized *knowledge & awareness* and mental health. Almost all organizations provided general information on preventive measures for COVID-19. Very few provided links to governmental online COVID-19 self-assessment tools similar to that advocated by the Public Health Agency of Canada [31] or addressed the pandemic’s implications on treatment. Only the Quebec Cancer Coalition and Colorectal Cancer Canada mentioned implications on screening [27,30]. 

*Supportive resources* provided by the included cancer organizations concentrated on three areas of patients’ overall wellbeing: financial, mental, and physical health. Concerning financial health, cancer organizations either offered available funds themselves or referred patients to governmental response funds. The Canadian Breast Cancer Network’s website refers patients with cancer to the Canada Emergency Response Benefit, provincial support funds for business owners, and an assessment tool to determine if an individual qualifies for particular public aid funds [32]. Childhood Cancer Canada’s website provides eligibility criteria and information regarding the COVID-19 Emergency Fund for Children, Adolescents, and Young Adults with Cancer [33]. In terms of physical health, the supportive resources provided by organizations consisted of workout videos recorded by cancer support centers, such as those provided by Kidney Cancer Canada, or written nutritional guidance, such as the pamphlet provided by Colorectal Cancer Canada [30,34]. The mental aspect of a patient’s wellbeing was the most provided for by a majority of the included cancer organizations. General mental health was recognized by the online presence of groups such as the Canadian Cancer Society, which refers patients and caregivers to a mental health hotline, and contains recorded webinars addressing fear of cancer recurrence, living with isolation, and end of life during COVID-19 [35]. For example, Childhood Cancer Canada’s website incorporates a webinar addressing interpersonal difficulties within families of childhood cancer patients or survivors during the pandemic [33].

## 4. Discussion

Current research shows that cancer patients worldwide have experienced deferrals, modifications, and cancellations of necessary cancer treatment procedures. A survey, commissioned by the Dutch Federation of Cancer Patients Organisations, demonstrated that 30% of 5302 cancer patients reported changes (e.g., adjustment, delay, and/or discontinuation) in their oncological treatment between March and April 2020 [36]. Another study, using a web-based questionnaire to assess the impact of COVID-19 between 21 April and 8 May 2020 on 356 centers from 54 countries, reported that 88% of centers faced challenges in delivering care during the pandemic, and 55% had reduced services as part of their mitigation strategy [37]. A survey by the Canadian Cancer Survivor Network of 1243 cancer patients reported that 54% had their appointments canceled, postponed, or rescheduled between 22 May and 10 June 2020 [38]. A UK-based mathematical model, using 10-year net survival data (2008-17), indicated that a 1-month delay could lead to an additional 1412 lives lost and that a 6-months delay would result in 9280 additional lives lost [39]. 

The insufficient emphasis on *cancer control* in the identified CPGs raises concerns regarding the future of cancer prevention and screening services. In Canada, national restrictions have led to temporary suspensions of cancer screening programs [40] resulting in increased cancellations and postponements of routine diagnostic procedures. Worldwide, the rates of newly diagnosed cancers have fallen dramatically [41]. A report by the Epic Health Research Network on aggregated data for 2.7 million patients from 190 hospitals across 23 states in the US showed that, between 20 January and 21 April 2020, the weekly volumes of routine screening appointments for breast, colon, and cervical cancers fell by 94%, 86%, and 94% respectively, compared to corresponding weekly averages from the previous three years [42]. Delays in breast, colorectal, lung, and oesophageal cancer diagnoses would result in an estimated 3291 to 3621 additional deaths, and 59,204 to 63,229 additional years of life lost [43]. An anticipated concern centers on managing the screenings backlog and patient hesitancy in returning for routine on-site preventive care services. Therefore, resumption of safe, active cancer surveillance needs to be endorsed by CPGs that emphasize cancer control.

A review of guidelines on cancer care and control issued, in response to the COVID-19 pandemic, by recognized European and international cancer organizations in developed countries suggested that these might not be applicable to certain emerging low- and middle- income countries [44], as diagnostic and treatment delays predate the pandemic in these limited-resource settings. The authors highlighted the need for equitable guidelines and strategies that are suited for resource-limited countries. 

During the pandemic, patients with cancer have also struggled to access a spectrum of resources and activities that normally help them manage their physical, mental, and financial wellbeing. For example, activities that were once in-person at cancer wellness centres, such as physical therapy, adapted yoga, or nutritional support, are no longer available with the same accessibility as they were before the pandemic. In a survey, conducted by Colorectal Cancer Canada (April–May 2020) with 57 respondents, 30% reported their inability to maintain a healthy lifestyle during the pandemic, with individual responses emphasizing a need for exercise and nutrition webinars [30]. In a survey distributed by the Quebec Cancer Coalition, respondents stated similar needs for nutrition and physical requirements, with comments largely centering on pain management [27]. To address the enormous strain on patients with cancer, numerous cancer organizations provided mental health resources, from in-person hotlines, articles about coping with stress, to video webinars led by psychologists, and clinical social workers and educators. In their responses to the Quebec Cancer Coalition and Colorectal Cancer Canada surveys, patients with cancer voiced all too clearly their feelings of being “abandoned”, “forgotten”, “suffering from isolation”, and “dying alone” [27,30]. Of the 592 respondents in the former survey, 67% indicated that they were anxious or experienced worsening of existing mental health difficulties because of the pandemic [27]. In the latter survey, 63% of the 57 respondents rated their stress levels as higher than usual due to COVID-19 [30]. Given the global economic hardship brought about by the pandemic, the untoward financial consequences experienced by patients with cancer have been exacerbated. An online survey of 539 renal cell carcinoma patients across 14 countries found that 59% of respondents doubted that their retirement and savings assets would cover the cost of treatment and 23% “did not feel in control of their financial situation” [45]. 

CPGs pertaining to the management of pediatric cancer during the pandemic were scarce. Although SARS-CoV-2 infections appear to affect children less severely than adults, those with certain underlying medical conditions (i.e., asthma, immunosuppression, etc.) might be at an increased risk for developing COVID-19 symptoms [46]. Almost one third of pediatric patients were admitted to the intensive care unit following a COVID-19 hospitalization [47]. To the best of our knowledge, the MSSS in Quebec was the only agency that established guidelines and recommendations to manage pediatric oncology patients during the pandemic at every level of their prioritization system, emphasizing that all pediatric cancer care and/or control services should be maintained with no interruptions [22].

There will be inevitable updates to the reviewed CPGs and resources, given the constantly evolving circumstances surrounding the pandemic. Hence, the component checklists, Table 4 and Table 5, may become outdated over time. It is also possible that we missed other cancer-related CPGs that were uploaded on the web or circulated internally. We might have also overlooked other critical descriptive components. Notwithstanding, those described in Table 2 and Table 3, reflect major cancer-related services that were covered by the CPGs up until mid-November 2020. In addition, social media platforms used by patient advocacy groups, such as Facebook, Instagram, and Twitter, were not explored but such outlets may have provided pertinent resources to cancer communities during the COVID-19 pandemic [48]. However, we assumed that any information delivered to the public through social networks would have been communicated by the cancer organizations on their websites. It is also difficult to verify the reliability of information presented through social media. Moreover, the reviewed Canadian CPGs need not be applicable to other countries, specifically those with limited resources.

## 5. Conclusions

COVID-19 has imposed provisional changes in cancer care and control protocols at provincial and territorial levels in Canada. Canadian public health and medical professional agencies acted swiftly at the beginning of the pandemic, issuing and updating cancer care and control guidelines. They will be under pressure to provide the same rapid response as the pandemic evolves in the coming months. The availability of these guidelines and resources not only assuages the frustrations of physicians and anxieties of patients with cancer, but also fosters a trusting relationship between healthcare providers and patients.

## Figures and Tables

**Table 1:** Clinical practice guidelines on cancer care and control pre- and during COVID-19 by province.

Province	Public Health Agency	Pre-COVID-19 [Reference Number]	During COVID-19 [Reference Number]
Alberta	Alberta Health Services	Cancer Guidelines for Health Professionals [2]	COVID-19 Scientific Advisory Group Rapid Response Report [3] Cancer Treatment Prioritization Framework for Systemic Therapy, Radiation Therapy, and Supportive Care [4] Re-Testing of Immunocompromised Cancer Patients post-COVID-19 Diagnosis [5]
British Columbia	BC Cancer Agency	Cancer Management Guidelines [6]	Provincial Clinical Cancer Management Guidelines in Pandemic Situation [7] BC Cancer Tumour Group Specific Prioritization and Mitigation Recommendations during COVID-19 Pandemic [8]
Manitoba	Cancer Care Manitoba	Cancer Management Guidelines [9]	COVID-19 Important Information for Health Care Providers Regarding Cancer Patients [10] Information for Healthcare Providers on COVID-19 [11] Clinical Guidelines for Prioritizing Cancer Services in Manitoba during COVID-19 Pandemic [obtained via personal communication] [12]
Newfoundland and Labrador	Eastern Health	Cancer Care Guidelines [13]	Not Available
Nova Scotia	Nova Scotia Health Authority	Cancer Management Guidelines [14]	COVID-19 Oncology Protocol [obtained via personal communication] [15] COVID-19 Hub: Cancer Care [16] Perioperative and Interventional Radiology Services During the COVID-19 Pandemic [17]
Ontario	Ontario Health and Cancer Care Ontario	Cancer Treatments [18]	COVID-19 Supplemental Clinical Guidance for Patients with Cancer [19] Pandemic Planning Clinical Guideline for Patients with Cancer [20]
Québec	Ministère de la Santé et des Services sociaux	Recommandations concernant le diagnostic et le traitement du cancer dans les établissements du Québec [21]	Directives COVID-19 du ministère de la Santé et des Services sociaux [22]
Saskatchewan	Saskatchewan Cancer Agency	Clinical Practice Guidelines [23] Follow Up Guidelines [24]	Not Available

Yukon, Nunavut, and Northwest Territories, as well as Prince Edward Island did not have enough COVID-19 cases to justify issuing “during COVID-19” CPGs. New Brunswick used other provinces’ CPGs.

**Table 2:** Overview by theme of the components and characteristics of Canadian clinical practice guidelines for cancer care and control during the COVID-19 pandemic.

Theme	Components	Characteristics and Type of Information Provided
**Cancer care**	Broad-spectrum treatment	General prioritization criteria for anticancer treatment based on risk of tumour progression, as well as treatment form (i.e., adjuvant, curative, neoadjuvant, palliative), performance/symptom status, efficacy, need, age, capacity, comorbidities, and tumour stage, among other factors
	Cancer-specific treatment	Prioritization criteria for anticancer treatment by cancer site
	Radiation-specific treatment	Prioritization criteria to triage patients with cancer to radiation therapy
	Systemic-specific treatment	Prioritization criteria to triage patients with cancer to systemic therapy
	Surgical-specific treatment	Prioritization criteria to triage patients with cancer to surgical procedures
	Palliative-specific care	Prioritization criteria to triage patients with cancer to palliative care (i.e., symptom management, psychosocial support, end of life care)
	Treatment for newly diagnosed patients	Guidelines and/or recommendations to manage treatments for newly diagnosed patients with cancer
	Treatment delay	Estimated wait time (days, weeks, or months) to target treatment (i.e., systemic, surgery, radiation therapy) particular to each prioritization level
**Cancer control**	Broad-spectrum screening	General prioritization criteria for screening procedures based on form (i.e., diagnostic, routine, genetic, follow-up) and type (i.e., CT scan, MRI, mammogram) of screening procedure, as well as screening urgency to diagnose and/or treat, among other factors
	Routine-specific screening	Prioritization criteria to triage individuals to routine screening procedures (i.e., mammograms, Pap smear tests, HPV tests)
	Diagnostic imaging	Prioritization criteria to triage individuals to diagnostic imaging procedures (i.e., computed tomography, magnetic resonance imaging, positron emission tomography)
	Genetic screening	Prioritization criteria to triage individuals to genetic screening procedures for oncological disorders
	Screening delay	Estimated wait time (days, weeks, or months) to screening procedures (i.e., mammograms, Pap smear tests, human papillomavirus tests, computed tomography lung scans) particular to each prioritization level or category
**Cancer care & control for COVID-19** **patients**	General information	Information (i.e., diagnosis, symptoms, treatments, modes of transmission) related to COVID-19 disease and/or SARS-CoV-2 infection
	Precautionary measures	Information about personal preventive measures (i.e., practicing good hygiene, maintaining physical distancing, wearing masks) and/or departmental mitigation measures (i.e., remote consultations when possible, minimizing additional visitors in waiting rooms)
	Treatment management	Guidelines and/or recommendations to manage anticancer treatment procedures for patients with cancer who tested positive for COVID-19
	Screening management	Guidelines and/or recommendations to manage cancer screening operations (i.e., colorectal, cervical, breast cancer screening) for individuals who tested positive for COVID-19

**Table 3:** Overview by theme of the components and characteristics of Canadian resources for cancer care and control during the COVID-19 pandemic.

Theme	Components	Characteristics and Type of Information Provided
**Knowledge & awareness**	General information	Public health information about COVID-19 (i.e., definition, symptoms, diagnosis, treatment) and/or links to relevant, external resources
	Preventive measures	Protective practices related to SARS-CoV-2 (i.e., handwashing, wearing masks, social distancing advice) and/or the meaning of being immunocompromised and at an increased risk of developing worse COVID-19 symptoms compared to healthy individuals
	Self-assessment tool	Link(s) to provincial and/or federal online COVID-19 self-assessment tools including online series of inquiries into whether an individual has experienced any COVID-19 symptoms, travelled outside of Canada recently, had close contact with a symptomatic person, and/or travelled recently in the past 14 days
**Cancer care &** **control during COVID-19**	Implications on treatment	Effects of COVID-19 on in-patient and/or out-patient cancer treatments including visitation changes and alterations in treatment timelines and/or information on putative changes to treatment if a patient were to test positive for COVID-19 during treatment
	Implications on screening	Effect of the COVID-19 pandemic on access to cancer screening (i.e., regular screening, pre-treatment surveillance, post-treatment screening, self-sampling)
**Supportive resources**	Physical health resources	Access to articles, links, and/or webinar series along with professional advice (i.e., oncology-specific nutrition advice) and resources (i.e., exercise webinars) relating to physical health of patients with cancer
	Financial resources	Internal (i.e., donor-supported financial programs) and/or external financial resources (i.e., links to applications for employment insurance sickness benefits, Canada’s Emergency Response Benefit, donor-sourced financial support, Canada’s COVID-19 Economic Response Plan) for patients with cancer
	Mental health resources	Access to articles, external links, lecture series, online support groups, and/or webinars relating to mental health implications (i.e., anxiety, fear, isolation) for patients with cancer and their caregivers

**Table 4:** Checklist of Canadian clinical practice guidelines amid the COVID-19 pandemic by theme, descriptive components, and provincial agencies.

Provincial Agency [References]	Cancer Care (i.e., Treatment)								Cancer Control (i.e., Screening)					Cancer Care and Control for Patients with COVID-19			
	Broad-Spectrum	Cancer-Specific	Radiation-Specific	Systemic-Specific	Surgical-Specific	Palliative-Specific	Newly Diagnosed Patients	Delay	Broad-Spectrum	Routine-Specific	Diagnostic Imaging	Genetic	Delay	General Information	Precautionary Measures	Treatment Management	Screening Management
**Alberta Health services [3,4,5]**	✓		✓	✓	✓	✓		✓						✓	✓	✓	
**British Colombia Cancer [7,8]**	✓	✓	✓	✓		✓	✓	✓	✓	✓	✓	✓	✓		✓	✓	
**Manitoba Cancer Care [10,11,12]**	✓		✓	✓	✓											✓	
**Nova Scotia Cancer Care [15,16,17]**	✓		✓	✓		✓	✓	✓						✓	✓	✓	
**Ontario Health Cancer Care Ontario [19,20]**	✓	✓	✓	✓	✓	✓	✓	✓	✓	✓	✓	✓	✓	✓	✓	✓	
**Quebec- Ministère de la Santé et des Services sociaux [22]**	✓	✓	✓	✓	✓		✓	✓	✓	✓	✓		✓	✓	✓	✓	

Yukon and the Northwest Territories have not modified their oncological practice, thus their cancer care and control clinical practice guidelines have not been changed. Nunavut operating under the Ottawa-Bafin Island program infers treatment using Ontario cancer guidelines. New Brunswick and Prince Edward Island refers to guidelines issued by other Canadian agencies including Ontario, British Columbia, and Quebec.

**Table 5:** Checklist of resources and recommendations amid the COVID-19 pandemic by theme, descriptive components, and organization.

Society	Knowledge & Awareness			Cancer Care & Control during COVID-19		Supportive Resources		
	COVID-19 Information	SARS-CoV-2 Preventive Measures	Self-Assessment Tool	Implications on Treatment	Implications on Screening	Physical Health Resources	Financial Resources	Mental Health Resources
All.Can Canada	✓	✓		✓		✓		✓
Bladder Cancer Canada	✓	✓						
Breast Cancer Action Kingston	✓	✓						
Canadian Association of Psychosocial Oncology	✓	✓						✓
Canadian Breast Cancer Network	✓	✓	✓	✓			✓	✓
Canadian Cancer Society	✓	✓	✓	✓		✓	✓	✓
Canadian Cancer Survivor Network	✓	✓		✓		✓		✓
Childcan							✓	✓
Childhood Cancer Canada Foundation	✓	✓						✓
The Chronic Myelogenous Leukemia Society of Canada	✓	✓						
Colorectal Cancer Canada	✓	✓			✓	✓		✓
Fondation Virage pour le soutien au cancer						✓		
Hearth Place Cancer Support Centre								✓
Island Kid’s Cancer Association	✓	✓						✓
Kidney Cancer Canada	✓	✓		✓		✓		
Lung Cancer Canada	✓	✓		✓				✓
Myeloma Canada	✓	✓						
Ontario Parents Advocating for Children with Cancer	✓	✓	✓				✓	
Quebec Breast Cancer Foundation	✓	✓		✓	✓			
Rethink Breast Cancer	✓	✓						✓
The Cedars Cancer Foundation	✓	✓						
West Island Cancer Wellness Centre	✓							✓

Of the 187 cancer organizations screened, 22 societies provided COVID-19 information specific to cancer patients. The societies’ webpages were last accessed on 4 November 2020. Hyperlinks were generated for quick access to the COVID-19 information provided by each society.
